# Association between mask-associated dry eye (MADE) and corneal sensations

**DOI:** 10.1038/s41598-022-23994-0

**Published:** 2023-01-28

**Authors:** Takashi Itokawa, Yukinobu Okajima, Hiroko Iwashita, Kakisu Koji, Takashi Suzuki, Yuichi Hori

**Affiliations:** 1 Department of Ophthalmology, Toho University Graduate School of Medicine, 6-11-1, Omori-Nishi, Ota-Ku, Tokyo, 143-8541 Japan; 2 Ishizuchi Eye Clinic, Niihama, Ehime Japan

**Keywords:** Biomarkers, Diagnostic markers, Predictive markers

## Abstract

To determine the risk of mask-associated dry eye (MADE), we investigated the fluorescein tear break-up time (FBUT), ocular surface temperature and blood flow, along with corneal sensitivity, in mask wearers. We enrolled 60 mask wearers (mean age, 27.1 ± 5.2 years) and then measured FBUT, corneal temperature and conjunctival blood flow without wearing masks (no mask), with masks, and with taped masks. We defined MADE as the condition in which dry eye symptoms appeared and the FBUT with mask was less than 5 s. The FBUT with a mask was significantly shorter compared to the no mask and taped mask groups (*P* < 0.01 and *P* < 0.05). The corneal temperature difference and conjunctival blood flow difference were significantly higher after wearing a mask than after wearing a taped mask (*P* < 0.01). Of the 60 subjects, 13 were diagnosed with MADE. Pain sensitivity and the Ocular Surface Disease Index (*P* < 0.05 and *P* < 0.01) were significantly higher in the MADE group, with the FBUT without masks (*P* < 0.05) significantly shorter than in the non-MADE group. MADE may be associated with corneal hypersensitivity. Wearing masks decreased FBUT and increased ocular surface temperature and blood flow. Taping the top edge of masks prevented these changes. Fitting masks properly may reduce MADE risk.

## Introduction

Several studies have reported deterioration of the ocular surface along with increased dry eye symptoms during the COVID-19 pandemic^1,2^. One of the causes for the increased dry eye in patients has been suggested to be wearing a mask, which has been referred to as mask-associated dry eye (MADE)^3,4^. MADE has been thought to be caused by airflow leaking from the top edge of the mask^5^. Worldwide, the frequency of MADE has been reported to be approximately 8–30%^1,6–8^.

It has been reported that continuous airflow stimulation could be a cause of dry eye^9,10^. For example, an increase in dry eye symptoms has been noted during obstructive sleep apnea syndrome (OSAS) treatment, which is referred to as continuous positive airway pressure (CPAP), as the airflow leaking from the CPAP mask affected the tear fluid conditions on the ocular surface^11–13^. Harrison et al. reported that compared to the baseline conditions, airflow leakage associated with CPAP treatment led to irritation and problems with tear film stability due to tear film evaporation in these patients^11^.

Furthermore, it has been reported that ocular stimulations due to airflow currents can affect not only tear film stability and dry eye symptoms but also ocular surface temperatures and conjunctival blood flows^12,14,15^. A previous study demonstrated that the trigeminal nerve can perceive changes in corneal temperature^16^. Furthermore, decreases in corneal temperature are associated with the detection of dryness symptoms, with corneal temperature increases suppressing the secretion of tear fluid^16,17^. The corneal temperature varied depending on the flow rate of the air stimulation and the temperature of the airflow. When the airflow rate was used as the stimulus, increases in the corneal temperature were dependent on the temperature of the airflow used during the stimulation^15^. Furthermore, in a previous study by Chen et al. that evaluated airflow stimulation and subsequent changes in bulbar conjunctival blood flow, the authors reported that airflow stimulation significantly increased bulbar conjunctival blood flow due to the associated efferent parasympathetic responses^14^. Therefore, our hypothesis is that airflow that leaks from the upper edge of the mask may have an effect on not only tear film stability and ocular symptoms, but also corneal temperature and conjunctival blood flow.

Since MADE does not always occur when wearing masks, it can be assumed that changes in corneal sensitivity that are perceived as changes in ocular surface parameters could also potentially have an effect on the occurrence of MADE. Situ et al. evaluated the sensitivity of subjects with dryness symptoms and reported that compared to normal subjects, the study subjects exhibited hyperesthesia^18^. Recently, several researchers reported that subjects with short break-up time (BUT) dry eye often had a discrepancy between their subjective symptoms and objective signs, with these subjects having a significantly higher corneal pain sensation than normal subjects, although the tactile sensation was similar between the two groups^19,20^. To the best of our knowledge, there have yet to be any studies that have investigated these types of changes in ocular surface parameters other than tear film stability and staining grade in subjects when using a mask or in changes in corneal sensitivity that are associated with MADE. The aim of our current study was to investigate the effects of the airflow that leaks from the top edge of masks during normal breathing on tear film stability, corneal and conjunctival temperature and blood flow, as well as to further examine the differences in ocular surface parameters, including corneal sensitivity with and without MADE.

## Results

### Demographic characteristics of the subjects

Table 1 presents the demographic characteristics of 60 healthy volunteers (30 females and 30 males) who participated in this study. All participants wore face masks for over 6 h a day during the COVID-19 pandemic. The average age was 27.1 ± 5.1 years old. There were 31 subjects who wore a soft contact lens (SCL). In subjects without SCL wearers (n = 29), corneal and conjunctival temperature and conjunctival blood flow in the condition of without mask, taped mask, and mask were not significantly different (*P* > 0.05, unpaired t-test) compared to SCL wearers (n = 31). The same trend obtained for corneal tactile sensitivity, corneal pain sensitivity and ocular surface disease index (OSDI).

**Table 1:** Demographic data.

Parameters	Subjects (n = 60)
Age (years)	27.1 ± 5.1 (25.7–28.4)
Gender (male : female)	30:30
Non SCL wearer : SCL wearer	29:31
Heart rate (bpm)	71.1 ± 10.1 (68.1–72.8)
MABP (mmHg)	85.0 ± 14.7 (82.4–89.7)
Body temperature (°C)	36.7 ± 0.4 (36.7–36.8)
Corneal tactile sensitivity (cm)	5.2 ± 0.9 (5.0–5.5)
Corneal pain sensitivity (cm)	3.3 ± 1.4 (3.0–3.7)
OSDI scores	8.3 ± 9.2 (5.9–10.7)
FBUT (sec)	6.4 ± 3.1 (5.8–7.2)
Corneal temperature (°C)	34.33 ± 0.49 (34.20–34.45)
Bulbar conjunctival temperature (°C)	34.39 ± 0.47 (34.27–34.51)
Bulbar conjunctival blood flow (MBR)	162.6 ± 40.7 (152.1–173.1)

The data are presented as the average ± deviation (95% confidence interval). *SCL* soft contact lens; *MABP* mean arterial blood pressure; *OSDI* Ocular surface disease index; *FBUT* fluorescein tear break-up time; MADE, mask-associated dry eye.

### Changes in the parameters of the ocular surface while wearing a face mask

We compared the ocular surface parameters in the same subject during three types of conditions: (1) when the mask was removed (no mask), (2) when the mask was attached without taping (mask), and (3) when the mask was attached after taping the top edge (taped mask).

### Fluorescein tear break-up time (FBUT)

**Figure 1:**
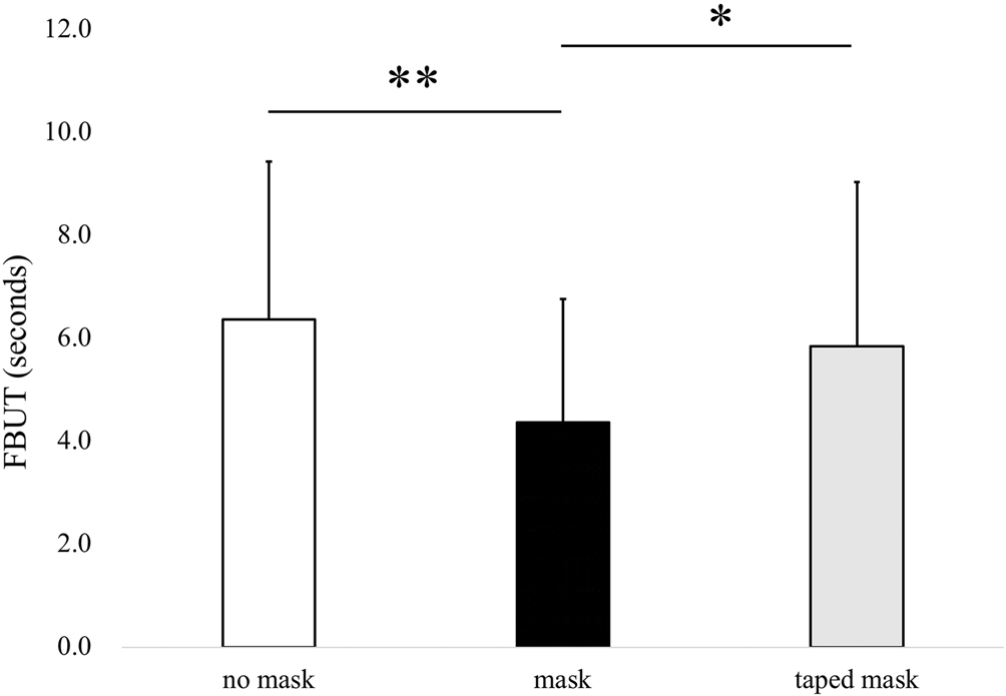
Comparison of the FBUT during the no mask, mask and taped mask conditions. Data are presented as the mean ± standard deviation. The asterisks indicate significant differences during these conditions (*P < 0.05 and **P < 0.01, Tukey HSD test). FBUT, fluorescein tear breakup time.

The FBUTs of the no mask, mask, and taped mask groups were 6.4 ± 3.1, 4.4 ± 2.4 and 5.8 ± 3.2 s, respectively. Although the FBUT value for the mask group was significantly shorter than that found for the no mask and taped mask groups (*P* < 0.01 and *P* < 0.05, respectively, Tukey HSD test), the values for the no mask and taped mask groups were not significantly different (Fig. 1).

### Corneal and bulbar conjunctival temperature

**Figure 2:**
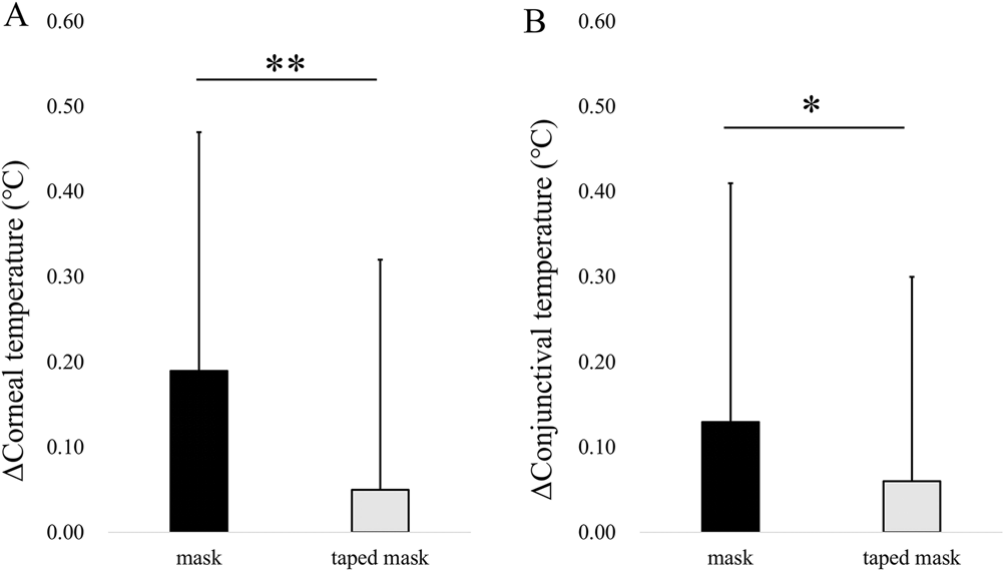
Comparison of the difference in the corneal (**A**) and bulbar conjunctival (**B**) temperatures between the no mask and mask or taped mask groups. The asterisks indicate significant differences during these conditions (*P < 0.05 and **P < 0.01, paired t-test).

**Figure 3:**
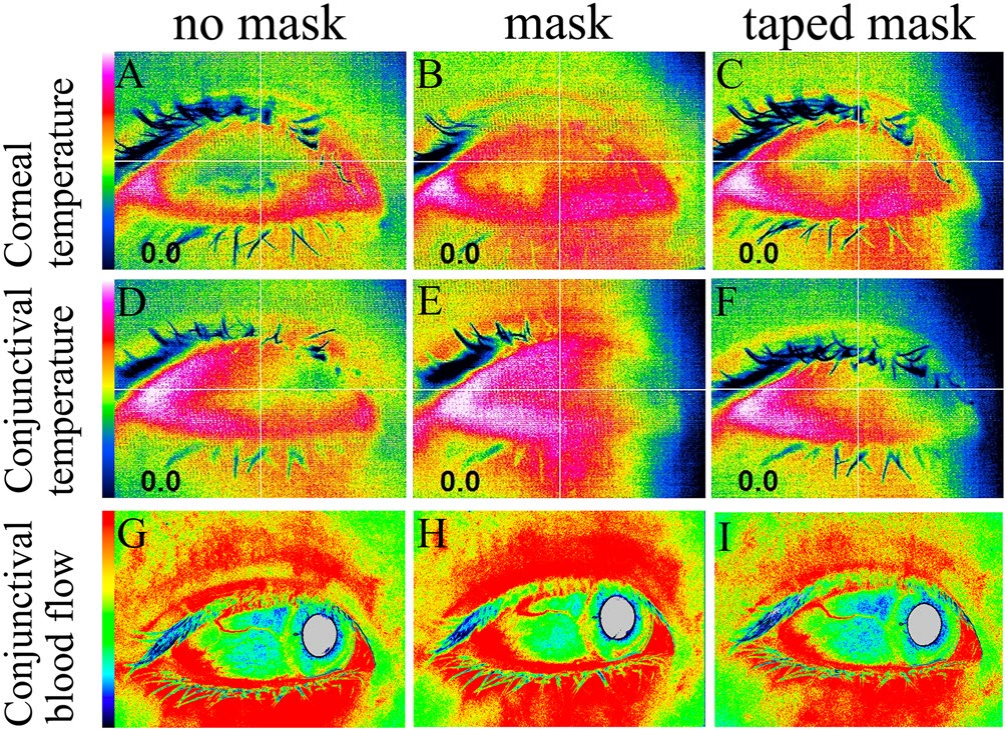
Representative images of corneal and conjunctival temperature (**A** to **F**) and conjunctival blood flow (**G** to **I**). Corneal temperature with no mask (33.85 ℃, **A**), mask (34.31 ℃, **B**) and taped mask (34.08 ℃, **C**). Conjunctival temperature with no mask (34.08 ℃, **D**), mask (34.47 ℃, **E**) and taped mask (34.19 ℃, **F**). Conjunctival blood flow with no mask (191.3, **G**), mask (256.5, **H**) and taped mask (178.4, **I**). Images were made using Adobe photoshop elements 2018 software.

The difference in the corneal temperature (Δcorneal temperature) between the no mask and mask groups (0.19 ± 0.28 °C, mask minus no mask) was significantly higher than the difference observed between the no mask and taped mask groups (0.05 ± 0.27 °C, taped mask minus no mask) (*P* < 0.01, paired t-test, Fig. 2A). The difference in the bulbar conjunctival temperature (Δconjunctival temperature) between the no mask and mask groups (0.13 ± 0.28 °C) was significantly higher than that found between the no mask and taped mask groups (0.06 ± 0.24 °C) (*P* < 0.05, Fig. 2B). Representative images of the conjunctival and corneal temperature with no mask, mask, and taped mask were shown in Fig. 3A–F.

### Conjunctival blood flow

**Figure 4:**
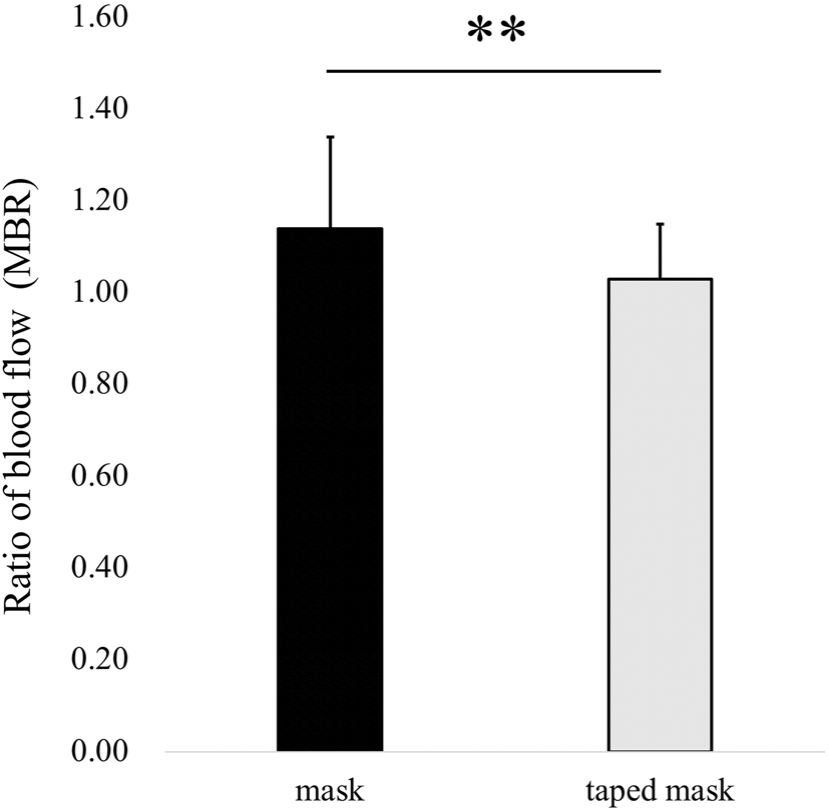
Comparison of the bulbar conjunctival blood flow rates between the no mask and mask or taped mask groups. The asterisks indicate significant differences during these conditions (**P < 0.01, paired t-test). MBR, mean blur rate.

The change in the bulbar conjunctival blood flow between the no mask and the mask conditions (1.14 ± 0.20) was significantly higher than that observed between the no mask and the taped mask conditions (1.03 ± 0.12, *P* < 0.01, Fig. 4). Representative images of the conjunctival blood flow with no mask, mask and taped mask were shown in Fig. 3G–I.

### Comparison of ocular surface parameters between MADE and non-MADE

**Figure 5:**
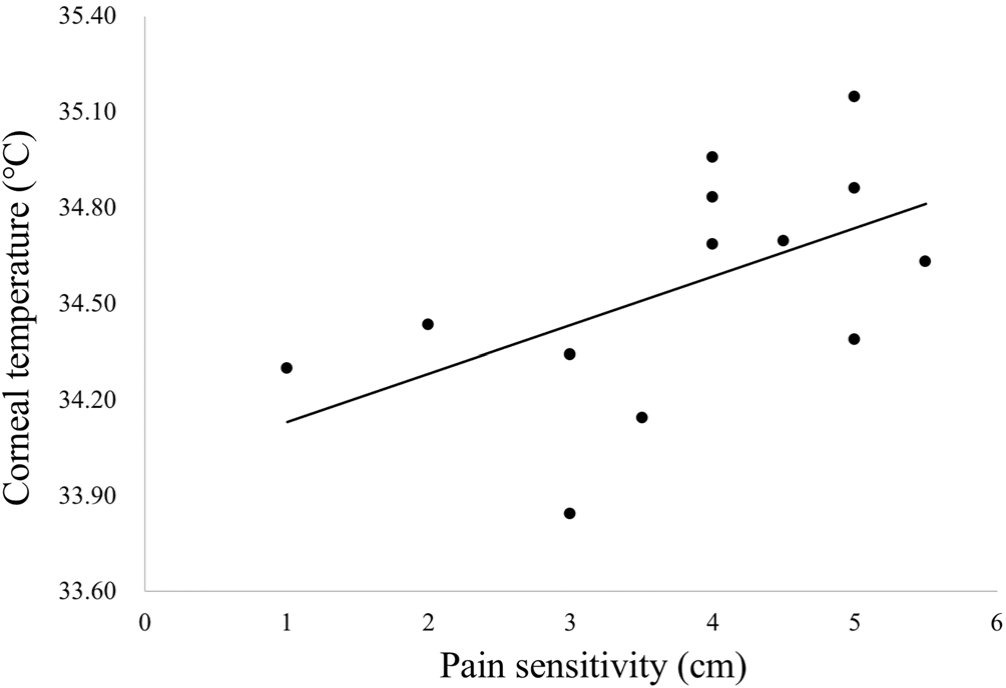
Association between corneal temperature with mask and pain sensitivity in MADE group (n = 13). Positive correlation was shown between the corneal temperature with mask and pain sensitivity (r = 0.5925, *P* < 0.05, Spearman’s rank correlation coefficient).

In this study, we defined MADE as the condition in which dry eye symptoms, i.e., dryness, pain and discomfort, appeared or worsened in conjunction with the shortening of the FBUT to less than 5 s when wearing a mask. Of the 60 eyes, 13 (21.7%) eyes were classified as MADE. The systemic and ocular surface parameters without and with a mask were compared between the MADE and non-MADE groups (Table 2). In the MADE group, the Ocular Surface Disease Index (OSDI) was significantly higher (15.3 ± 11.3 vs. 6.4 ± 7.6 s, *P* < 0.01, unpaired t-test), and the FBUT without (4.5 ± 2.1 vs. 6.9 ± 3.1 s, *P* < 0.05) and with a mask (2.8 ± 1.3 vs. 4.8 ± 2.5 s, *P* < 0.01) was significantly shorter than that observed for the non-MADE group. When adjusted for the value of corneal tactile sensitivity, the pain sensitivity of the MADE group was significantly higher (3.8 ± 1.3 vs. 3.2 ± 1.4 mm, *P* < 0.05, ANCOVA) than that observed for the non-MADE group, which indicated that the subjects in the MADE group were significantly more hypersensitive to corneal pain. The conjunctival (r = 0.6593; *P* < 0.05, Spearman’s rank correlation coefficient) and corneal (r = 0.5925; *P* < 0.05, Fig. 5) temperature with mask in MADE group was significantly correlated with pain sensitivity, but non-MADE group was not recognized significant correlation in both parameters. Conjunctival blood flow was not recognized these correlations in MADE and non-MADE group.

**Table 2:** Comparison of the results between the MADE and non-MADE groups.

	MADE (n = 13)	Non-MADE (n = 47)	*P* value
Age (years)	27.5 ± 5.1 (24.4–30.5)	27.0 ± 5.3 (25.4–28.5)	0.7606
Gender (male : female)	5 (38.5%) : 8 (61.5%)	25 (53.2%) : 22 (46.81%)	0.4190
SCL wearer	9 (69.2%)	22 (46.8%)	0.1474
Heart rate (bpm)	69.8 ± 10.9 (63.2–76.4)	70.6 ± 8.9 (68.0–73.2)	0.7675
MABP (mmHg)	80.5 ± 11.6 (73.4–87.5)	87.6 ± 14.3 (83.4–91.8)	0.1063
Body temperature (°C)	36.8 ± 0.2 (36.7–36.9)	36.7 ± 0.4 (36.6–36.8)	0.4026
Corneal tactile sensitivity (cm)	5.1 ± 1.1 (4.4–5.8)	5.2 ± 0.9 (5.0–5.5)	0.6677
Corneal pain sensitivity (cm)	3.8 ± 1.3 (3.0–4.6)	3.2 ± 1.4 (2.8–3.6)	**0.0410**
OSDI scores	15.3 ± 11.3 (8.5–22.1)	6.4 ± 7.6 (4.1–8.6)	**0.0014**
FBUT (sec)	4.5 ± 2.1 (3.3–5.8)	6.9 ± 3.1 (6.0–7.8)	**0.0138**
With mask	2.8 ± 1.3 (2.0–3.6)	4.8 ± 2.5 (4.1–5.5)	**0.0060**
Corneal temperature (°C)	34.30 ± 0.46 (34.02–34.58)	34.34 ± 0.50 (34.19–34.48)	0.8285
With mask	34.56 ± 0.36 (34.34–34.77)	34.51 ± 0.43 (34.38–34.64)	0.7523
Conjunctival temperature (°C)	34.44 ± 0.42 (34.19–34.70)	34.37 ± 0.48 (34.23–34.52)	0.6370
With mask	34.60 ± 0.39 (34.37–34.83)	34.50 ± 0.43 (34.37–34.62)	0.4346
Conjunctival blood flow (MBR)	174.2 ± 44.9 (147.1–201.3)	159.4 ± 39.3 (147.8–170.9)	0.2496
With mask	189.9 ± 54.4 (157.0–222.8)	181.5 ± 46.0 (167.9–194.9)	0.5769

The data are expressed as the average ± deviation (95% confidence interval). Parameters in the MADE and non-MADE groups. The data are presented as the average ± deviation (95% confidence interval). *SCL*, soft contact lens; *MABP* mean arterial blood pressure; *OSDI* Ocular Surface Disease Index; *FBUT*, fluorescein tear breakup time. Bold values indicate significant differences.

## Discussion

During the COVID-19 pandemic, there has been a greatly increased frequency of face mask wearing, with the subsequent increased rate of dry eyes potentially associated with this mask wearing^3,4^. In our current study, we investigated the changes in tear film stability (FBUT), ocular surface temperature, and conjunctival blood flow that were observed in subjects wearing masks. We additionally compared ocular surface parameters, including corneal sensitivity in subjects with and without MADE. When wearing a mask, tear film stability was decreased, and there was an increase in corneal and conjunctival temperature and conjunctival blood flow. These parameters returned to their baseline values (no mask) when the upper gap of the mask was closed with tape. Subjects with MADE had a significantly worse OSDI score and tear film stability, including the FBUT, with or without a mask. Furthermore, compared to the subjects without MADE, our findings also interestingly showed that the corneal sensitivity evaluation in subjects with MADE found the presence of hyperalgesia.

In our current study, the prevalence of MADE was 21.7% (13/60). Several other previous cross-sectional survey studies have reported a prevalence of MADE in 8–31% of participants^1,6–8^. However, to date, there have been no standard criteria established for diagnosing MADE. In our current study, we diagnosed MADE based on both the aspects of the symptoms and tear film stability (FBUT < 5 s). In addition, our current study recruited subjects who wore a face mask for more than 6 h a day. Thus, differences in the diagnostic criteria and population differences could account for the varying prevalence of MADE that was observed.

Previous studies have also reported that the factors that were potentially associated with causing MADE included being female and having a history of dry eye symptoms before the pandemic^1,6–8^. In our current study, although we did not determine the differences to be significant, there was a higher rate of females in the MADE group (61.5%), while males were dominant in the non-MADE group (53.2%). In this study, we recruited healthy volunteer who did not complain of dry eye symptom and were not attending a clinic. Criteria of dry eye was defined by the Asia Dry eye Society^21^, and subjects with ocular symptom and FBUT < 5 s were diagnosed with dry eye. Of all 60 eyes, 25 eyes had FBUT < 5 s, and 10 of 25 eyes were in the MADE group. Although our current study did not specifically ask about a previous history of dry eye, the scores for the dry eye questionnaires (OSDI score) were significantly higher than those of the non-MADE group, and there was a significantly lower FBUT in subjects without a mask. Therefore, these findings suggested that the MADE group in this study included many patients in the preclinical stage of dry eye.

Compared to the non-MADE group, subjects with MADE were hypersensitive to corneal pain, although the tactile sensitivity was the same in both groups. This finding suggests that the MADE groups had corneal hyperalgesia. Perceptual studies using the Belmonte esthesiometer have reported that there are two types of sensation in dry eye, i.e., hypoesthesia^22,23^ and hyperesthesia^18,24,25^. Studies using the Cochet-Bonnet esthesiometer reported that hyperalgesia was associated with the short BUT type, which was indicative of high pain sensitivity, although the tactile sensation was found to be the same^19,20^. Perceptual changes are caused by reduced tear film stability and a lowered corneal threshold due to inflammation of the ocular surface^26,27^. Mastropasqua et al. reported that wearing a mask for more than 6 h significantly increased the dendritic cell density and HLA-DR, which represent inflammatory markers, with these changes not observed in those subjects who only wore face masks less than 3 h per day^2^. These findings suggest that wearing a mask for more than 3 h increases the inflammatory response in a time-dependent manner^2^. Furthermore, MADE may be caused by inflammation and nerve abnormalities associated with the airflow that leaks from the mask, and subsequently affects the ocular surface. Therefore, these findings appear to indicate that these effects can lower the sensory threshold and lead to changes in ocular surface parameters that will subsequently cause discomfort.

The mechanism responsible for the worsening of the tear film stability while wearing a mask was believed to be the same as that found when wearing a CPAP for the treatment of OSAS, which has been shown to induce tear film instability due to rapid evaporation of tear film by the leaking airflow^11–13^. In our current study, the tear film stability decreased from 6.4 to 4.4 s while wearing a mask, although the tear film stability recovered at 5.8 s after taping the upper edge of the mask. Thus, improper wearing of a mask can induce MADE, due to decreases in the tear film stability associated with the airflow that exits from the top edge of the mask. Fan et al. reported that approximately 40% of mask wearers had improper fittings and an odds ratio for MADE due to improper fitting of 1.4, with a higher incidence of MADE associated with improper mask fitting^6^. Aksoy et al. reported that wearing a mask with a gap at the upper edge of the mask for at least 8 h led to the tear film becoming unstable, although the condition of the tear film stability returned to normal after taping down the upper edge of the mask^28^. Nair et al. also reported this phenomenon when using an N95 mask with a significant correlation in the changes in the differences between the subjective symptoms and FBUT before and after the use of an N95 mask^29^. In these two studies, subjects wore masks for 8 h. However, in our current study, we observed tear film instability after wearing a mask for 15 min. Thus, this is the first report to demonstrate the presence of tear film instability from a relatively early phase when wearing a mask.

In addition to our results showing that airflow leaking from the upper edge of the mask caused a decrease in tear film stability, we also found that several other ocular surface parameters were changed, including corneal and bulbar conjunctival temperatures. When wearing a mask, our results showed that the corneal and bulbar conjunctival temperatures increased by 0.19 and 0.13 °C, respectively. However, this increase in the temperature was not observed after taping the face mask at the upper edge. Nosch et al. evaluated the same airflow velocities but with different temperatures and found that there was a significantly increased corneal temperature when the airflow was warmer than the corneal temperature^15^. Based on our current study results, we speculated that since a person’s breath is normally warmer than the corneal temperature, this is responsible for causing corneal and bulbar conjunctival temperatures to increase in this study. Bourcier et al. reported that the mean threshold for heat stimulation was 0.21 °C in subjects under 40 years old^23^. Parra et al. reported a decreased tearing rate when corneal temperature reached 36.0 °C^17^. Since we found that the corneal temperature changed when wearing a mask, this suggests that there should be changes similar to those found for the heat stimulation threshold. Therefore, the results of our present study indicate that the temperature changes that were caused by the wearing of a mask were not high enough to cause a decrease in the ability to secrete tear fluid. Moreover, it is possible that MADE subjects experience a perceptual abnormality that is perceived as a temperature change that would not usually be recognized as being uncomfortable in normal subjects.

Another ocular surface change was the bulbar conjunctival blood flow. Some researchers have reported a significant increase in bulbar conjunctival blood flow in patients with dry eye and in contact lens wearers, which causes chronic or subclinical ocular inflammation^30,31^. Chen et al. reported that bulbar conjunctival blood flow was significantly increased due to the perception of airflow stimulation by the sensory nerve ending of the trigeminal nerve, with the response carried out by the efferent neural pathway that innervates the ocular surface^14^. In our current study, we also found that when wearing a mask, there was a significant increase in the bulbar conjunctival blood flow due to stimulation by the airflow leaking out of the mask during breathing compared to that observed after taping the mask at the upper edge. Although a previous study^14^ investigated the response to cold air stimulation when conducted at room temperature, which used air that was colder than the corneal temperature, in our current study we found that stimulation by the airflow associated with warm breath that leaks out of the mask increased the bulbar conjunctival blood flow as well.

Perceptual threshold of healthy volunteers who have symptom was lower for mechanical stimulation by airflow and they become hyperesthesia^32^. Teson et al. reported that subjects with hyperesthesia have possibility in a preclinical stage of dry eye development, the inflammation is responsible for generating the hyperesthesia and the presence of symptom without any other ocular surface alterations^32^. We speculate that the MADE group had become hyperalgesia, and MADE onset due to tear film instability, inflammation by wearing mask more than 6 h, thermal stimulation by warm breath and mechanical stimulation by airflow from the edge of mask. Several researchers reported that in humans, range of corneal temperature was between 33.0 and 36.0 ℃ in the condition of general environment and variations after eye opening was less than 1 ℃^33–37^. Bourcier et al. reported normal eye and dry eye with hypoesthesia perceive the decreased temperature by less than 0.01 and 0.12 ℃, and the increased temperature by more than 0.21 and 0.32 ℃^23^. They also found that cold, thermal, chemical and mechanical threshold had correlated each other^23^. Short BUT dry eye subjects with hyperalgesia show the correlation between pain sensitivity and symptom^20^. In this study, the corneal temperature with mask in MADE group was significantly correlated with pain sensitivity, but non-MADE group was not recognized significant correlation. In the MADE group, the corneal temperature increased as the pain sensitivity specifically increased. In other words, the more severe the hyperalgesia, the more the corneal temperature increased due to warmed airflow leakage from the top edge of the mask.

There were some limitations to our current study. First, we recruited healthy volunteers with and without the use of SCL. Although previous studies have reported that wearing contact lenses does not affect MADE^6,7^, it could be possible that wearing an SCL might alter the bulbar conjunctival microvascular morphology and microcirculation^30^. In the future, we are planning to investigate the differences in blood flow and temperature in SCL wearers with and without MADE. Second, in this study, we did not measure Schirmer test, tear meniscus height, which are indicators of tear fluid volume, tear break-up pattern, and also observe corneal and conjunctival epithelial damage or blepharitis. These are important in understanding the pathogenesis of MADE and classification MADE into dry eye subtype. Third, there is a certain number of mask wearers who become aware of their better symptom after wearing a mask^7^. We assume that better symptom after wearing mask caused by an increase in tear fluid volume due to reflex secretion, which is stimulated the ocular surface by the airflow leaking from the top edge of mask. In this study, because we compared non-MADE to MADE, we did not investigate the difference between mask wearers with better and worse symptom. In the future, we need to investigate the difference in corneal perception, ocular surface temperature and conjunctival blood flow between those whose subjective symptoms better and worse. Forth, mask wearing is recommended in Japan to prevent infection due to the COVID-19 pandemic. Therefore, it was not possible to have subjects stop wearing a mask over a certain period of time as a wash out, and thus, this could have potentially had an effect on the results of our current study.

In conclusion, factors associated with MADE include corneal hyperalgesia, worse subjective symptoms and tear film instability prior to wearing a mask. Furthermore, as the presence of a gap at the upper edge of a mask can cause tear film instability, increases in corneal and bulbar conjunctival temperatures and bulbar conjunctival blood flows, it is possible that the cause of MADE could be due to the perception of these changes in the ocular surface. However, it is possible to prevent these changes by simply taping the upper edge of the mask. Although there has been a decrease in the presence of COVID-19 in the population, thereby leading to a decrease in the necessity to wear masks, the possibility cannot be ruled out that new infectious disease outbreaks could once again force people to have to wear masks in the future. Current results suggest that masks need to be properly worn by closing the upper edge of the mask to prevent MADE risk.

## Methods

### Subjects

We enrolled 60 healthy subjects (30 females and 30 males; 27.1 ± 5.2 years; range, 21–38 years) who wore masks for more than 6 h a day. The exclusion criteria included a history of allergic keratoconjunctivitis, Meibomian gland dysfunction, ocular injuries, infections, keratitis, and ocular surgery. This study protocol was approved by the Ethics Committee of the Faculty of Medicine, Toho University (# A22011_A20115), and registered with UMIN000045398. All of the procedures used conformed to the tenets of the Declaration of Helsinki. Informed consent was obtained from all subjects after a detailed explanation of the nature and possible consequences of the study.

### Corneal and conjunctival bulbar temperature

Corneal and temporal bulbar conjunctival temperatures were measured using an ocular surface thermographer (TG1000; Toomey Corporation, Nagoya, Japan) on the right eye of each of the subjects. Subjects were asked to close their eyes for 5 s, and then measurements were performed after opening their eyes^31,33,34,38^. Corneal temperature was analyzed quantitatively in the central 4 mm diameter of the cornea. Temporal bulbar conjunctival temperature was analyzed quantitatively within a 4 mm circle that was 1 mm away from the temporal corneal limbus^34^. We conducted the corneal and conjunctival temperature measurement in two minutes. For systemic parameters, we also measured body temperature. To make representative image, we used a computer graphics program (Adobe Photoshop elements 2018; Adobe System, Inc., San Jose, CA).

### Bulbar conjunctival blood flow

Temporal bulbar conjunctival blood flow was measured using the laser speckle flowgraphy-ocular anterior segment (LSFG-OAS, Softcare, Fukuoka, Japan) procedure, which is a modified version of the LSFG for skin blood flow measurement^34^. The mechanism of LSFG has been previously published^34,39^. Briefly, a speckle pattern forms due to interference related to scattered rays reflected by erythrocytes in the blood vessels of the region of interest. The speckle pattern, which varies in accordance with the velocity of the erythrocyte, was evaluated as the mean blur rate (MBR) using LSFG analysis software (version 3.8.0.4, Softcare, Fukuoka, Japan). Although the MBR represented the relative blood flow velocity, according to a previous study, the MBR and other blood flow measurement techniques are represented by the absolute values that were correlated^40,41^. All subjects were placed at an angle of approximately 40 degrees to the nasal side, and the measurement was then conducted for 4 s without blinking. The measurement location of temporal bulbar conjunctival blood flow was the same as that measured for the temperature. The bulbar conjunctival blood flow was analyzed quantitatively by 60 pixels in a circle that was 20 pixels away from the temporal corneal limbus. The blood flow measurement was conducted five consecutive times, with the average of the central three data points then analyzed as the MBR^34^. We conducted the bulbar conjunctival blood flow measurement in a minute. For the systemic parameters, we also measured the heart rate (beats/min [bpm]) and the mean arterial blood pressure (MABP) (mmHg). MABP was calculated from the following formula: diastolic blood pressure + (systolic blood pressure − diastolic blood pressure)/3.

### Fluorescein tear break-up time (FBUT)

The fluorescein tear break-up time (FBUT) was measured using a fluorescein test strip (Fluores Ocular Examination Test Paper, Ayumi Pharmaceutical Co., Tokyo, Japan). The central top of the strip was gently touched to the central lower lid margin. After natural blinks, the subjects were then asked to gently close this eye followed by briskly opening of the eye^42^. FBUT was defined as the time until observation of the first break in the tear film during the opening of the eyes. Measurements were repeated 3 times, with the average of the data used as the FBUT.

### Corneal tactile and pain sensitivity

Corneal sensitivity was measured using a Cochet-Bonnet esthesiometer. The Cochet-Bonnet esthesiometer was a 6.0 cm long flexible monofilament nylon thread with a diameter of 0.12 mm^43^. The thread was pressed on the central cornea at a perpendicular angle. Because the shorter the nylon thread was, the greater the stiffness of the thread became, the thread was shortened from 6.0 cm at 1.0 cm intervals. When the tactile sensation was first felt, the thread was lengthened by 0.5 cm and checked again to determine the tactile sensation. Pain sensation was further shortened from the value of tactile sensation, with the pain sensation determined in the same way as the tactile sensation^19,20^.

### Subjective symptoms

Subjective symptoms when not wearing masks were evaluated using the Japanese version of the Ocular Surface Disease Index (OSDI) questionnaire^44^. The OSDI contains 12 questions about the field of ocular symptoms, vision-related functions and limitations, and environment triggers. The subject’s answers ranged from 0 (none of the time) to 4 (all the time) with regard to the frequency for each of the questions. The total score, which was included on a 0- to 100-point scale, was calculated using the following formula: OSDI = (sum of scores × 25)/(total number of answered questions)^45^.

With regard to the subjective symptoms that were related to the face mask, subjects were asked if the dry eye symptoms, i.e., dryness, pain and discomfort, were better, worse or stayed the same, while wearing a mask.

### Study protocol

This study was conducted between 3:00 and 7:00 pm. The date of the first registration was 27/04/2021 (UMIN000045398). The temperature (26.2 ± 1.1 °C) and humidity (37.4 ± 11.1%) in the measurement room were maintained at constant levels. SCL wearers were asked to discontinue the wearing of the SCL starting on the day before the measurement. All subjects used the same type of mask (Super Comfortable Mask, Unicharm). At first, 15 min after the subjects entered the examination room, we performed the initial measurements while the subject wore no mask. After these measurements, subjects were provided a mask that covered the mouth and nose but with a created gap of 1 cm in the upper part of the mask that allowed for air blowing upward from the mask onto the ocular surface. After resting for 15 min under these conditions, the measurements were performed again. Finally, the mask was repositioned so that it covered the mouth and nose with surgical tape applied to seal the superior part of the mask to prevent air blowing from the mask and going to the ocular surface. Under these conditions, subjects were asked to wait at rest for 15 min after which the measurements were once again performed. To minimize the effects of measurements on the ocular surface parameters while being examined under the above three conditions, the examinations were conducted in the order of FBUT, corneal and conjunctival temperatures, and conjunctival blood flow. During the measurements, the start of each measurement was synchronized with the exhalation timing, with the subjects instructed to repeat their breathing once every 2–3 s. Before the measurement of the ocular surface parameters, body temperature, MABP, heart rate, OSDI and subjective symptoms associated with wearing a mask were assessed. Corneal tactile and pain sensations were tested after all measurements were completed, due to their invasiveness.

### Statistics

We determined the sample size based on previous study, which in a FBUT of 8.8 and 10.06 s with and without taping the mask, indicating that at least 52 subjects were required for this study design (α = 0.05, power 80%). The data are expressed as the average ± deviation (95% confidence interval). Multiple comparisons were performed using the Tukey honestly significant difference (HSD) test when significant differences were identified across three conditions. Spearman’s rank correlation coefficient was used to assess the correlations between pain sensitivity and ocular surface temperature and conjunctival blood flow. A paired t-test was used to compare the changes in the parameters between the no mask and the mask or taped mask. The unpaired t-test and chi-squared test were used to compare the parameters between the non-MADE and MADE groups or non-SCL wearers and SCL wearers. Moreover, the analysis of covariance (ANCOVA) test was used to compare the pain sensation between the groups after adjusting for tactile sensations. *P* values of less than 0.05 were considered statistically significant. All analyses were conducted using JMP version 14 statistical analysis software (SAS Institute Inc., Cary, NC, USA).

### Ethical approval

This study protocol was approved by the Ethics Committee of the Faculty of Medicine, Toho University (# A22011_A20115), and the trial was registered with UMIN000045398.

## Data Availability

The data used to support the findings of this study are available from the first author, TI, upon reasonable request.
